# Evaluation of the Effects of Chia (*Salvia hispanica* L.) Leaves Ethanolic Extracts Supplementation on Biochemical and Hepatic Markers on Diet-Induced Obese Mice

**DOI:** 10.3390/antiox12051108

**Published:** 2023-05-17

**Authors:** Gabriela Maturana, Javiera Segovia, Claudio Olea-Azar, Ernesto Uribe-Oporto, Alejandra Espinosa, María Carolina Zúñiga-López

**Affiliations:** 1 Department of Inorganic and Analytical Chemistry, Faculty of Chemical and Pharmaceutical Sciences, University of Chile, Santiago 8380494, Chile; gamaturana@uchile.cl (G.M.); colea@uchile.cl (C.O.-A.); 2 Department of Medical Technology, University of Chile, Santiago 8380453, Chile; javierasegovia@uchile.cl (J.S.); ernesto.uribe@ug.uchile.cl (E.U.-O.); 3 Escuela de Medicina, Universidad de Valparaíso, Valparaíso 2340000, Chile

**Keywords:** *Salvia hispanica* L., leaf extracts, hepatic damage, obesity

## Abstract

Obesity is a significant health concern affecting 13% of the world’s population. It is often associated with insulin resistance and metabolic-associated fatty liver disease (MAFLD), which can cause chronic inflammation in the liver and adipose tissue. Obese hepatocytes show increased lipid droplets and lipid peroxidation, which can lead to the progression of liver damage. Polyphenols have been shown to have the ability to reduce lipid peroxidation, thereby promoting hepatocyte health. Chia leaves, a by-product of chia seed production, are a natural source of bioactive antioxidant compounds, such as cinnamic acids and flavonoids, which have antioxidant and anti-inflammatory properties. In this study, chia leaves’ ethanolic extracts of two seed phenotypes were tested on diet-induced obese mice to evaluate their therapeutic potential. Results show that the chia leaf extract positively affected insulin resistance and lipid peroxidation in the liver. In addition, the extract improved the HOMA-IR index compared to the obese control group, reducing the number and size of lipid droplets and lipid peroxidation. These results suggest that chia leaf extract may help treat insulin resistance and liver damage associated with MAFLD.

## 1. Introduction

An imbalance between calorie consumption and expenditure may produce obesity, a disease characterized by excessive adipose tissue accumulation and chronic inflammation of adipose tissue and the liver. In obesity, the secretion of inflammatory cytokines is enhanced, thus, promoting an inflammatory environment that alters glucose metabolism [1]. Plasmatic glucose concentration is controlled primarily by insulin and glucagon signaling in the liver, adipose tissue, and skeletal muscle [2]. These hormones regulate glucose plasmatic concentrations and have antagonistic effects. While insulin stimulates glucose uptake by adipose tissue and skeletal muscle, glucagon stimulates glucose release from the liver by glycogenolysis and gluconeogenesis. Evidence suggests that in the obese adipose tissue and liver, inflammation mediators, such as TNF-α, alter insulin signaling and disturb glucose homeostasis [3,4,5], triggering insulin resistance (IR) [1]. Under these altered conditions, liver-specific abnormalities, such as metabolic-associated fatty liver disease (MAFLD), may arise as a consequence of impaired insulin signaling. Since hepatic free fatty acid production increases in the obese state due to exacerbated de novo lipogenesis, the status of hepatic steatosis is triggered. Lipid droplets (LD) accumulate in the liver, and their lipid peroxidation (LPO) increases, worsening IR further and perpetuating the oxidative stress status that results in hepatocyte damage and loss of its function [6]. Damaged hepatocytes secrete pro-inflammatory cytokines, such as TNF-α and IL-6, among others, which result in the recruitment of macrophages, maintaining the inflammatory process and promoting the progression from MAFLD to steatohepatitis and then fibrosis [6].

Traditional treatment of obesity includes a low-carbohydrate diet, drugs that increase insulin sensitivity, and incorporating physical exercise, all strategies to restore proper energy balance. However, only a few patients systematically incorporate these lifestyle changes. The incorporation of fruits and vegetables in the diet is based on their richness in bioactive agents, such as polyphenols, known for their antioxidant properties. According to several studies, these compounds improve insulin sensitivity in the initial stages of establishing IR. In this regard, promising results have been published for a wide range of vegetal natural products [7,8,9]. Polyphenols may also exert regulatory effects at the cellular level, stimulating gene expression to modulate cellular metabolism [10,11,12], specifically obesity-related effects including a wide range of action and mechanisms, from reducing the expression of pro-inflammatory cytokines to regulating antioxidant enzyme action and improving glucose homeostasis [13]. Within the group of polyphenols with proven antioxidant effects, hydroxycinnamic acids could be mentioned, which significantly reduce the blood concentration of free fatty acids, triglycerides, insulin, and total cholesterol in obese animals fed a high-fat diet (HFD) [14]. In the same line, rosmarinic acid (RA), a hydroxycinnamic acid derivative, has been found to suppress lipid accumulation, enhance lipolysis, and downregulate adipogenic factors in human adipocytes [15]. RA and caffeic acid (CA), have recently been identified and quantified in chia leaf ethanolic extracts (CLEEs) [16].

Chia (*Salvia hispanica* L.) is an herbaceous plant belonging to the Lamiaceae family native to southern Mexico [17], widely known for its seeds and their nutritional value, as they possess high concentrations of α-linolenic acid [18,19,20,21,22], polyphenols, and amino acids [22]. Thus, the commercial cultivation of this plant is focused on seed production and yield. So far, there is a wide range of scientific work on the nutritional value of chia seeds and their effects on improving metabolic alterations associated with IR [8], while chia leaves have been scarcely studied even though they represent an important source of antioxidants with excellent health and commercial potential [23,24,25], exposing the necessity of exploring the relevance of other aerial parts, which have yet to be appreciated due to the lack of scientific studies. Amato et al. first reported the phenolic composition of chia leaf methanolic extracts by HPLC-ESI-MS, finding 34 metabolic compounds including hydroxycinnamic acids, such as CA and RA, and flavonoids, mainly luteolin and apigenin derivatives [25]. More recently, a study performed by Zúñiga et al. characterized eight chia leaf sequential extracts from two different seed phenotypes by UPLC-HRMS. This study also evaluated the in vitro antioxidant capacity (AC) and successfully identified and quantified the caffeic acid content (CAC) and rosmarinic acid content (RAC), finding that black chia ethanolic extracts had the highest AC on the chemical experiments, but that white chia ethanolic extract performed better when the cellular antioxidant activity was evaluated [16].

Following the lines of this previous research and continuing with the in vivo evaluation of ethanolic extracts, the main objective of the present work was to evaluate the effect of ethanolic extracts of white and black chia on the improvement in some serum markers related to alterations associated with obesity, along with evaluating the improvement in the levels of both steatosis and hepatic lipoperoxidation.

## 2. Materials and Methods

### 2.1. Cultivation, Sampling and Drying of Chia Plants

The plants used in this study were cultured at the experimental field INIA Intihuasi, belonging to the University of Chile, near La Serena city (Chile) (28°34′41.01″ S-70°47′52.62″ W). The culturing was carried out between January and June 2020 in triplicate for each phenotype (black and white seeds). The samples were taken to the laboratory within the same day, and then the leaves were separated, labeled, and dried at 35 °C in a stove until they reached a constant weight. After the drying process, the leaves were crushed with a mortar and stored at room temperature in darkness.

### 2.2. Extraction

CLEEs were obtained after successive exhaustive extractions with different organic solvents: hexane, dichloromethane, ethyl acetate, and ethanol, as reported previously [16]. Only the ethanolic extracts were used for biological experiments since they present the higher antioxidant capacity, CAC, and RAC for the series [16]. Each extraction was carried out until the matrix was completely exhausted. Between each extraction, the matrix was dried at room temperature before adding the new dissolvent. The final dried sequential extracts were obtained after concentration in a rotary evaporator at reduced pressure.

### 2.3. Animals and Diet

Weaned healthy males (C57BL/6J, four weeks old, 20 ± 2 g) were obtained from the Public Health Institute, Santiago, Chile. All mice were housed at a conventional facility in a temperature-controlled room with a 12 h:12 h light/dark cycle and were given access to their specific diet and water ad libitum. Six animals were randomly selected and fed a standard diet (10% fat, 20% protein, and 70% carbohydrates, D12450J, Research Diets, New Brunswick, NJ, USA) during the entire trial (Control Diet group; CD). After 12 weeks, this group was supplemented with distilled water. Parallelly, 18 57BL/6J male mice were fed with a high fat diet (HFD) (60% fat, 20% protein, and 20% carbohydrates, D12492, Research Diets, New Brunswick, NJ, USA) for 12 weeks. After that period, the mice were randomly divided into three groups with equal numbers of animals (*n* = 6); the first group was supplemented with distilled H_2_O (high fat diet group; HFD), the second was supplemented with an aqueous solution of B-CLEE at a dose of 50 mg/kg/day (HFD-BE), and the last group was supplemented with an aqueous solution of W-CLEE at a dose of 50 mg/kg/day (HFD-WE). All diets (CD and HFD) had the same micronutrient content.

**Figure 1:**
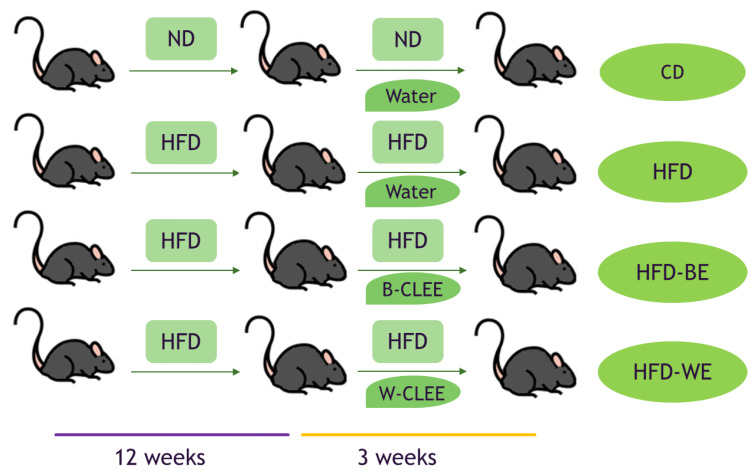
Feeding and supplementation protocol. ND, normal diet; HFD, high fat diet; B-CLEE, black leaf ethanolic extract; W-CLEE, white leaf ethanolic extract; CD, control diet group; HFD, HFD-fed non-supplemented group; HFD-BE. HFD-fed B-CLEE-supplemented group; HFD-WE, HFD-fed W-CLEE-supplemented group (*n* = 6 for each group).

CLEE supplementation was carried out once a day by means of a syringe by oral administration of the CLEE aqueous solution, ensuring all of it was consumed. The supplementation lasted three weeks without suppressing the HFD, as shown in Figure 1. All animals were cared for according to the Ethics Committee of the Chemical and Pharmacological sciences of the University of Chile (CBE-2019-07l; 19274-CyQ-UCh). At the end of the experimental period, mice were euthanized according to the protocol. Tissues (hepatic and epididymal fat) and blood samples were collected on the same day.

### 2.4. Intraperitoneal Glucose Tolerance Test

An intraperitoneal glucose tolerance test (IpGTT) was performed 1 w before euthanasia, after 6 h of fasting, by the administration of a 2 g/kg intraperitoneal glucose solution, and glycemia at 0, 15, 30, 60, 90, and 120 min after injection were measured, using tail blood samples. Blood glucose concentrations were measured in a commercial glucometer (Johnson and Johnson, New Brunswick, NJ, USA).

### 2.5. Weight Measurements

The total mass of every animal was measured before and after each supplementation to assess if the extracts affected body weight gain. The liver and epididymal fat were also measured for the same purpose.

### 2.6. Measurements of Serum Parameters

Serum was collected after blood centrifugation at 3500× *g* at 4 °C for 15 min and stored at −20 °C. Serum insulin concentrations were determined by a commercially available immunoassay specific for mice (Mercodia, Uppsala, Sweden). Measurements were performed the day after serum collection. Biochemical parameters were measured by dry chemistry technology (SPOTCHEM. EZ, Minneapolis, MN, USA). The HOMA-IR (homeostasis model assessment of insulin resistance) was calculated with Equation (1), as follows:

HOMA-IR = [fasting glucose (mg dL^−1^) × fasting insulin (µU mL^−1^)]/405, (1)

### 2.7. Histological Analysis of Liver

Liver samples were dehydrated and included in paraffin (Hisotec^®^), and then 5 µm cuts were carried out in a microtome to obtain histological cuts to be disposed of on a microscope slide for observation. Each slide was dyed with Mayer’s hematoxylin and 1% aqueous eosin for visualization in a clear field microscope. H&E liver images score was performed based on the criteria proposed by Liang et al. [26]. Macrovesicular steatosis and microvesicular steatosis were graduated based on the percentage of the total area of steatosis into the following categories: 0 (<5%), 1 (5–33%), 2 (34–66%), and 3 (>66%). The analysis was performed using Image J software (NIH).

### 2.8. Lipoperoxidation Labeling

To measure the LPO level and analyze lipid droplets, 4,4-difluoro-5-(4-phenyl-1,3-butadienyl)-4-bora-3a,4a-diaza-s-indacene-3-undecanoic acid (BODIPY^®^ 581/591 C11) (Invitrogen, Carlsbad, CA, USA) was used. This dye shifts from red to green upon oxidation. For hepatocyte isolation, 4–5 mm size samples were mechanically disaggregated using a Tissue Tearor homogenizer (Cole-Parmer, Vernon Hills, IL, USA), and purified using Ficoll (Sigma Aldrich, St. Louis, MO, USA) after centrifugation at 3000× *g* for 90 s. Freshly isolated hepatocytes were incubated with BODIPY 581/591 for 60 min at room temperature. Green and red fluorescence signals were captured by confocal microscopy (Nikon Spectral C2+ microscope, Tokyo, Japan). An acquisition protocol using simultaneous double wavelength excitation (laser lines Ex488/Em520 and Ex561/Em595nm, respectively) was used. Fluorescence intensity in both channels was quantified by FIJI (NIH, Bethesda, MD, USA) (National Institutes of Health, Bethesda, MD, USA).

### 2.9. Statistical Analysis

Data are presented as mean ± SEM. All data sets were tested for normality using Shapiro–Wilk and Kolmorov–Smirnov tests, and then significant differences between groups were examined using one-way ANOVA for repeated measures, followed by Tukey’s multiple comparison test; *p* < 0.05 was considered statistically significant (IC 95%). All statistical analyses were performed using GraphPad Prism 5.

## 3. Results

### 3.1. Weight Gain

The mass was measured to assess if the CLEEs affected body weight, liver weight, and epididymal fat weight gain. As shown in Table 1, all animals under HFD weighed similarly and were approximately 1.7 times the mean body weight of the CD at the end of the trial. Hence, CLEE supplementation appears not to affect overall body weight gain.

### 3.2. Tests to Assess Glucose Homeostasis

**Figure 2:**
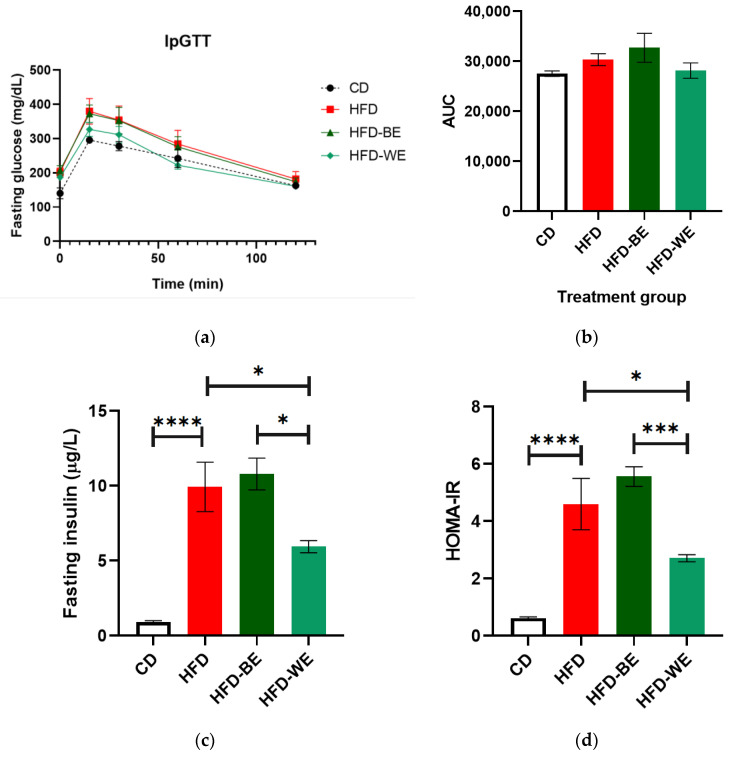
Tests to assess glucose homeostasis. (**a**) IpGTT curves; (**b**) area under the IpGTT curve (AUC); (**c**) fasting insulin and (**d**) HOMA-IR index. * Significant difference with a *p* < 0.05. *** significant difference with a *p* < 0.001. **** significant difference with a *p* < 0.0001. CD, control diet group; HFD, HFD-fed non-supplemented group; HFD-BE, HFD-fed B-CLEE-supplemented group; HFD-WE, HFD-fed W-CLEE-supplemented group (*n* = 6 for each group).

IpGTT was performed the day before euthanasia to establish if CLEEs had any effect on glucose tolerance. The results show no difference between HFD and HFD-BE (Figure 2a,b), but an improvement for HFD-WE, although it was not a statistically significant difference (*p* > 0.05) compared to HFD results.

The results show that the obesity model used is consistent with previous work because the HFD group presented higher fasting insulin values than the control group. However, treatment of obese animals with W-CLEE reversed the increase in insulin. A similar result to the previous one can be observed when performing the HOMA-IR calculation (Figure 2c,d).

### 3.3. Effect on Hepatic Damage Reversal

#### 3.3.1. Seric Parameters

**Figure 3:**
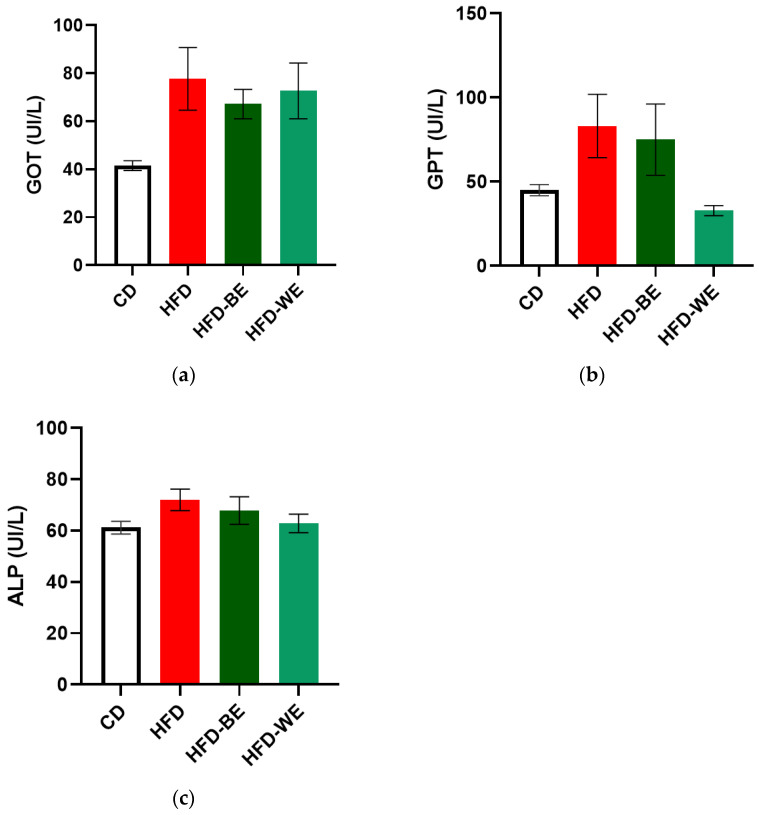
Hepatic profile for each treatment group. Serum concentration of (**a**) GOT; (**b**) GPT; (**c**) ALP. CD, control diet group; HFD, HFD-fed non-supplemented group; HFD-BE, HFD-fed B-CLEE-supplemented group; HFD-WE, HFD-fed W-CLEE-supplemented group (*n* = 6 for each group).

We measured classical serum markers to evaluate both the effect of the extracts on the reversal of liver damage and to provide evidence of a potential hepatotoxic effect of the extracts. High levels of glutamic-pyruvic transaminase (GPT) and glutamic-oxaloacetic transaminase (GOT) are markers of hepatic damage, especially for GPT [27]. As shown in Figure 3a,b, all the HFD-fed groups showed a higher concentration for both transaminases. HFD-BE and HFD-WE had the lowest transaminase concentration for GOT and GPT, respectively, among the three obese groups. Despite no statistical differences, a clear tendency for GPT was found, with the HFD-WE having a much lower value than HFD-BE and HFD for this transaminase. In the case of alkaline phosphatase (ALP) (Figure 3c), no significant differences were observed between groups, which is consistent with the fact that treatment with HFD for 3 months does not generate massive inflammatory liver damage [28], since liver inflammation could be triggered after a 34-week HFD period. Furthermore, CLEE supplementation did not affect either hepatocytes or bile ducts.

#### 3.3.2. Effect of CLEE on Liver Steatosis

**Figure 4:**
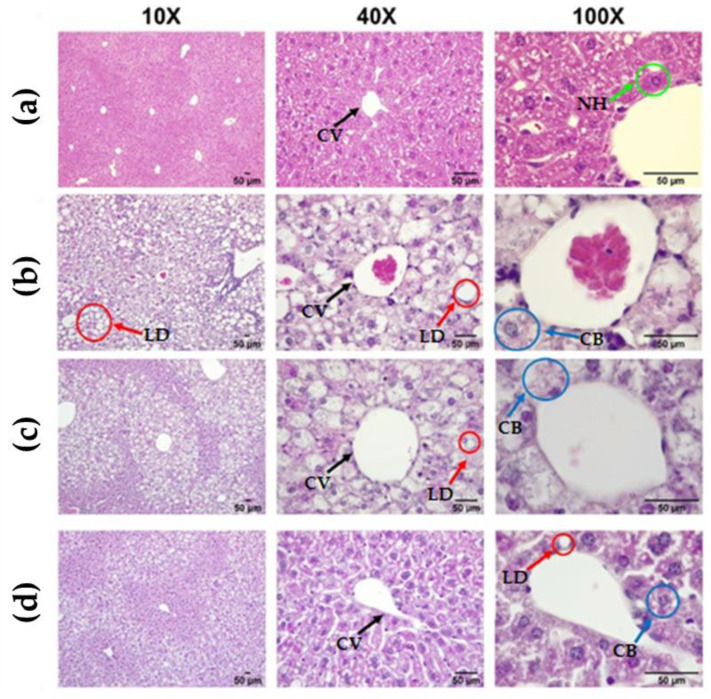
Representative bright field image of a H&E-stained liver section. From left to right: 10×, 40×, and 100× augmentation. (**a**) CD, (**b**) HFD, (**c**) HFD-BE, and (**d**) HFD-WE liver histology. CV: central vein; NH, normal hepatocyte; LD, lipidic droplets; CB, cellular ballooning. CD, control diet group; HFD, HFD-fed non-supplemented group; HFD-BE, HFD-fed B-CLEE-supplemented group; HFD-WE, HFD-fed W-CLEE-supplemented group (*n* = 6 for each group).

Until this point, the parameters evaluated to state the effect of CLEE supplementation on the reversal of hepatic damage show an improvement tendency, especially for the W-CLEE-supplemented group. Indeed, these data were complemented by more specific studies of liver histology and isolated hepatocytes. Figure 4 shows representative microscopies of hepatic tissue for every treatment group. Figure 4 shows representative images of the liver tissue of each group. When comparing the liver tissue of the HFD with the CD, the accumulation of hepatic LDs is evident, showing an apparent increase in the number and size of LDs in the form of macro- and microsteatosis. Six different fields were analyzed from each slide for each animal. The average was subsequently scored into categories based on macro- and microsteatosis percentage, hypertrophy, and inflammatory focus presence, as the scored system for NAFLD in mice was described [26]. Results show a score range of 0–1 for the CD group, 4–6 for the HFD group, 1–3 for the HFD-BE group, and 2–4 for HFD-WE (Supplementary Figures S1–S4).

Photographs acquired at 10× magnification show evident areas of steatosis (score 2/3, Liang et al. 2014 criteria [26]), which decreased with HFD-WE treatment (score 1/3). Treatment with HFD-WE partially reversed the macrosteatosis but did not affect the levels of microsteatosis (Figure 4 and Figures S1–S4).

#### 3.3.3. Lipidic Peroxidation State

**Figure 5:**
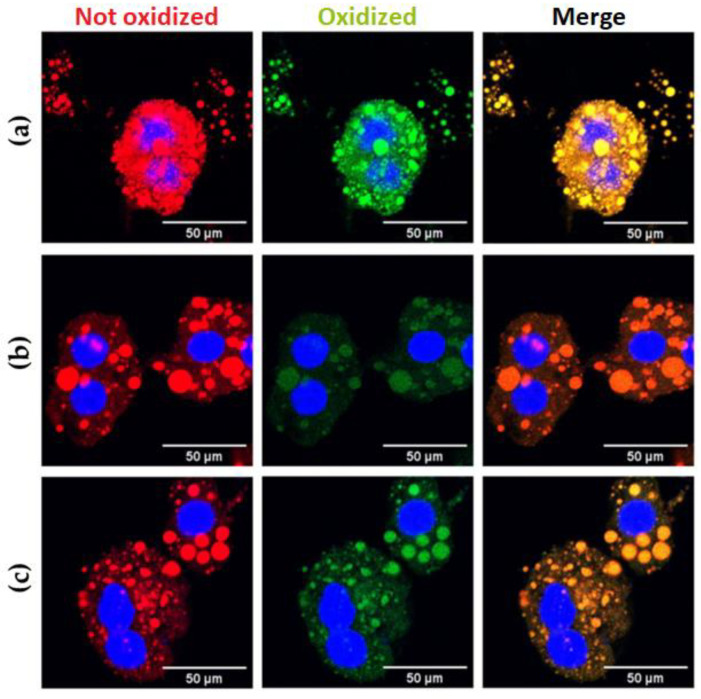
Hepatocyte lipidic droplets’ oxidation degree for (**a**) HFD, (**b**) HFD-BE, and (**c**) HFD-WE. Blue: hepatocytes’ nuclei marked with DAPI. Red: non-oxidated BODIPY emission. Green: oxidated BODIPY emission. HFD, HFD-fed non-supplemented group; HFD-BE, HFD-fed B-CLEE-supplemented group; HFD-WE, HFD-fed W-CLEE-supplemented group (*n* = 6 for each group).

The lipidic peroxidation (LPO) degree gives information on the actual cellular redox state of damaged tissue due to obesity-related steatosis. In order to evaluate the effect of both extracts on the degree of LPO in isolated hepatocytes, we used the fluorescent probe BODIPY-C11; upon oxidation, the fluorescence of this fluorophore shifts from red to green. Figure 5 and Figures S5–S7 shows representative images of isolated hepatocytes stained with BODIPY-C11, showing how the probe binds explicitly to LDs. The neutral, non-oxidized lipids are red, and the lipoperoxidation label is green. The treatments with the CLEE partially reversed both the number of total lipid droplets and the percentage of lipoperoxidation in these samples.

**Figure 6:**
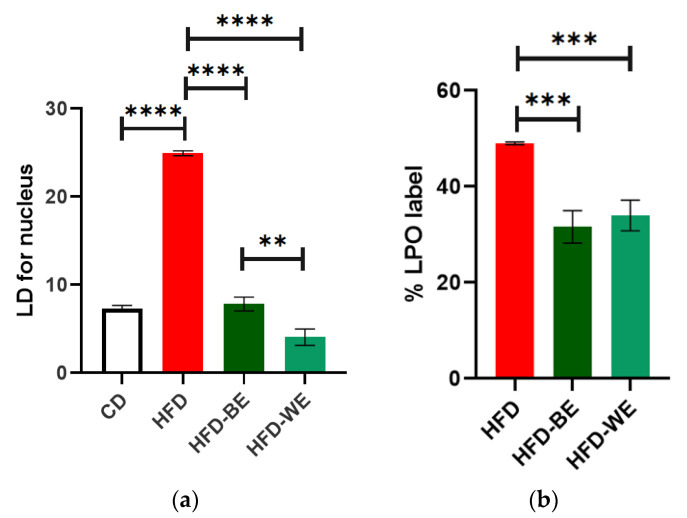
(**a**) Number of lipidic droplets for each nucleus for every treatment group, and (**b**) %LPO label of each HFD-fed group. ** Significant difference with a *p* < 0.01. *** Significant difference with a *p* < 0.001. **** Significant difference with a *p* < 0.0001. CD, control diet group; HFD, HFD-fed non-supplemented group; HFD-BE, HFD-fed B-CLEE-supplemented group; HFD-WE, HFD-fed W-CLEE-supplemented group (*n* = 6 for each group).

Both HFD-BE and HFD-WE showed a significatively decreases in the LD number of each hepatocyte compared with the HFD group (Figure 6a). The number of LDs in HFD-WE was lower than observed in HFD-BE. On the other hand, the ratio between green and red BODIPY-C11 labels, measured inside LDs, shows a significant decrease in the percentage of lipoperoxidation when animals were treated with B-CLEE and W-CLEE extract, with no difference between the lipoperoxidation levels between both treated groups.

## 4. Discussion

In this work, we evaluated the effects of two ethanol extracts obtained from chia leaves on the reversal of liver damage associated with MALFD. The results show that white chia extract has potential beneficial effects on reversing insulin resistance, lipid accumulation, and hepatic lipoperoxidation in MALFD.

### 4.1. IR-Related Parameters

As shown in Table 1, all HFD-fed groups have a final body weight statistically different to the CD-fed group, but no difference between HFD-fed groups was observed. It is important to notice that the first metabolic changes due to CLEE supplementation occur at a cellular level, which may not greatly impact a macroscopic measure, such as body, hepatic or epididymal fat weight. To assess the supplementation effect on these variables, a longer supplementation trial is needed.

Glucose homeostasis includes every metabolic process that helps to regulate blood sugar levels. As mentioned previously, obesity may cause IR, which occurs as a bodily response to chronic hyperinsulinemia and the interference of inflammatory mediators with downstream insulin signaling molecules [1]. Some useful tools to diagnose IR include the IpGTT test, AUC, HOMA-IR index, and fasting insulin serum concentration, among others. In this regard, data show (Figure 2) a clear difference between CD and all HFD-fed groups, but also an improvement tendency for HFD-BE and HFD-WE compared to HFD, especially for the W-CLEE-supplemented group.

Flavonoids are known for their health benefits, among which an improvement in glucose metabolism is one of the reasons these compounds represent an alternative to traditional IR treatment. As stated previously, the main compounds found in chia leaves are luteolin, apigenin, and CA and RA derivatives [16,25], Their presence in the extracts may be one of the reasons why W-CLEE supplementation had a significant decrease, especially for the HOMA-IR index and fasting insulin content (Figure 2) [29,30]. As reported previously, RA concentration was 24.9 ± 0.4 mg/g for W-CLEE and 21 ± 1 mg/g for B-CLEE, respectively, while CA concentration was 2.5 ± 0.3 mg/g and 2.80 ± 0.02 mg/g, respectively, which means that each B-CLEE-supplemented mouse consumed a mean of 0.051 ± 0.001 mg/day and 0.0013 ± 0.0006 mg/day of RA and CA, respectively, while W-CLEE-supplemented mice consumed a mean of 0.0594 ± 0.0003 mg/day and 0.0010 ± 0.0003 mg/day of RA and CA, respectively [16].

Comparing both phenotypes, W-CLEE supplementation had a much higher impact on all the parameters associated with glycemic homeostasis that were tested, even though the phenotypes are not that different in regard to their phenolic compositions. This may suggest the presence of other non-identified compounds that give birth to this different behavior. Furthermore, the data dispersion for HFD-WE was lower than for HFD or HFD-BE, which may mean that the effect of W-CLEE supplementation was more homogeneous than the B-CLEE supplementation effect. The difference in phenotype was also shown in the cellular antioxidant activity (%CAA) assay, with the W-CLEE having better performance in a biological medium [16,25].

### 4.2. Effect on Hepatic Damage Reversal

The serum levels of GOT, GPT, and ALP were determined for hepatic damage reversal assessment. As is shown in Figure 3, for all parameters tested, there is an improvement tendency for the HFD-BE and HFD-WE compared to the HFD, even if it is not statistically significant, especially for GPT and ALP (Figure 3b,c). The murine obesity model using HFD feeding produces alterations in the hepatic microanatomy characterized by hepatic steatosis. Macrosteatosis and microsteatosis are among the findings present. In our work, we observed that the livers of obese mice developed hepatic steatosis, as we have previously reported [31]. Using H&E histology, we could show how the HFD-BE and HFD-WE groups decreased the areas of micro- and macrosteatosis (Figure 4c,d). In order to assess microsteatosis quantitatively, we included the analysis of isolated hepatocytes. A similar tendency was found for the number and size of LDs (Figure 6). LPO is the oxidative degradation of lipids in cell membranes, which can lead to the formation of reactive oxygen species (ROS) and subsequent damage to cells. BODIPY-C11 can be used to measure this process by detecting the accumulation of oxidized lipids in cells and tissues. In Figure 6b, we show that the percentage of LPO in the isolated hepatocytes was lower within the LD from the groups, with no differences observed between the HFD-BE and HFD-WE. As discussed previously, HFD has been shown to induce liver steatosis and lipoperoxidation, both of which can contribute to liver damage and disease. LPO is the oxidative degradation of lipids in cell membranes, which can lead to the formation of ROS and subsequent accumulation of lipid peroxides, which are toxic to cells, inducing ferroptosis [32]. The improvement produced by chia leaf extracts may be due to the polyphenol content of chia leaves [16,25]. Polyphenols have a positive impact on the liver [31,33] by mechanisms that include their antioxidant activity (by the regulation of antioxidant enzyme activity), metabolic activity (lipogenesis diminution), and anti-inflammatory activity (by regulation of pro-inflammatory factors). Among cinnamic acids, CA and RA have been reported to have hepatoprotective activity [34] on animal models and stimulate liver regeneration after injury. These effects may be responsible for the dramatic fall in LD count (Figure 5) for both CLEE-supplemented groups compared to HFD. Again, HFD-WE had better results than HFD-BE, but even for the last, the LD count was comparable to the CD.

LPO is usually linked with a depleted membrane function [35] Antioxidants can act by upregulating the expression and activity of glutathione peroxidases (GPx), which can help to scavenge lipid peroxides using glutathione as a crucial cellular antioxidant, preventing the accumulation of oxidative stress in the cell. Polyphenols, such as quercetin, elevate glutathione concentration [36], which improves GPx activity, reducing LPO. A previous study by Liu et al. (2011) showed that the cinnamic acid-rich fraction of Chinese wild blueberry had an important inhibitory effect on triglyceride synthesis [37]. Since the chemical polyphenolic compositions of B-CLEE and W-CLEE do not differ greatly [16], there may be other key factors in the improvement in hepatic overall health that have not yet been found, but given the vast amount of scientific work on polyphenols and IR and MAFLD, these compounds may be responsible for some of the improvement in liver damage due to an HFD.

## 5. Conclusions

Based on the present research, chia leaf extract has the potential to improve insulin resistance and reduce lipoperoxidation in the liver. In addition, recent studies have shown that chia leaf extract contains bioactive compounds, such as polyphenols and flavonoids, with antioxidant and anti-inflammatory properties. These findings suggest that chia leaf extract may have therapeutic potential for preventing and treating insulin resistance and liver damage associated with MAFLD. However, further research is needed to determine the optimal dosage and duration of treatment and potential side effects.

## Figures and Tables

**Table 1:** Body, liver, and epididymal weight measurements for all groups studied.

Treatment Group	Body Weight (g)	Liver Weight (g)	Epididymal Fat Weight (g)
CD	28 ± 2	1.1 ± 0.1	1.7 ± 0.1
HFD	48 ± 3 ****	2.0 ± 0.3 *	1.9 ± 0.5
HFD-BE	49 ± 5 ****	2.2 ± 0.8 **	1.8 ± 0.5
HFD-WE	48 ± 2 ****	1.8 ± 0.4	2.1 ± 0.2

Body weight: **** significant difference between treatment groups (*p* < 0.0001). Liver weight: * significant difference between CD and HFD (*p* < 0.05); ** significant difference between CD and HFD-BE (*p* < 0.01). CD, control diet group; HFD, HFD-fed non-supplemented group; HFD-BE, HFD-fed B-CLEE-supplemented group; HFD-WE, HFD-fed W-CLEE-supplemented group (*n* = 6 for each group).

## Data Availability

Data is contained within the article or supplementary material.

## Supplementary Materials

The following supporting information can be downloaded at: https://www.mdpi.com/article/10.3390/antiox12051108/s1. Figure S1: Representative bright field image of an H&E-stained liver section. Images are representative of each animal from the control group (n = 6). A: Upper panel shows 10× and bottom panel shows 40× magnification, respectively. B: Calculated score for steatosis expressed out of a 12 total score. Figure S2: Representative bright field image of an H&E-stained liver section. Images are representative of each animal from the HFD group (n = 6). A: Upper panel shows 10× and bottom panel shows 40× magnification, respectively. B: Calculated score for steatosis expressed out of a 12 total score. Figure S3: Representative bright field image of an H&E-stained liver section. Images are representative of each animal from the HFD-BE group (n = 6). A: Upper panel shows 10× and bottom panel shows 40× magnification, respectively. B: Calculated score for steatosis expressed out of a 12 total score. Figure S4: Representative bright field image of an H&E-stained liver section. Images are representative of each animal from the HFD-WE group (n = 6). A: Upper panel shows 10× and bottom panel shows 40× magnification, respectively. B: Calculated score for steatosis expressed out of a 12 total score. Figure S5: Representative confocal images of BODIPY-c11-stained isolated hepatocytes. Images are representative of each animal from the HFD group (n = 6). 40× magnification. Figure S6: Representative confocal images of BODIPY-c11-stained isolated hepatocytes. Images are representative of each animal from the HFD-BE group (n = 6). 40× magnification. Figure S7: Representative confocal images of BODIPY-c11-stained isolated hepatocytes. Images are representative of each animal from the HFD-WE group (n = 6). 40× magnification.

## References

[1] Gregor M.F., Hotamisligil G.S. (2011). Inflammatory Mechanisms in Obesity. Annu. Rev. Immunol..

[2] Aronoff S.L., Berkowitz K., Shreiner B., Want L. (2004). Glucose Metabolism and Regulation: Beyond Insulin and Glucagon. Diabetes Spectr..

[3] Hotamisligil G.S., Peraldi P., Budavari A., Ellis R., White M.F., Spiegelman B.M. (1996). IRS-1-Mediated Inhibition of Insulin Receptor Tyrosine Kinase Activity in TNF-α- and Obesity-Induced Insulin Resistance. Science.

[4] Hotamisligil G.S., Arner P., Caro J.F., Atkinson R.L., Spiegelman B.M. (1995). Rapid Publication Increased Adipose Tissue Expression of Tumor Necrosis Factor-α in Human Obesity and Insulin Resistance. J. Clin. Investig..

[5] Hotamisligil G.S., Shargill N.S., Spiegelman B.M. (1993). Adipose Expression of Tumor Necrosis Factor-α: Direct Role in Obesity-Linked Insulin Resistance. Science.

[6] Tateya S., Kim F., Tamori Y. (2013). Recent Advances in Obesity-Induced Inflammation and Insulin Resistance. Front. Endocrinol..

[7] Donado-Pestana C.M., Moura M.H.C., de Araujo R.L., Santiago G.D.L., Barros H.R.D.M., Genovese M.I. (2018). Polyphenols from Brazilian native Myrtaceae fruits and their potential health benefits against obesity and its associated complications. Curr. Opin. Food Sci..

[8] Fadwa E.-O., Amssayef A., Eddouks M. (2022). Antihyperglycemic and Antidyslipidemic Activities of the Aqueous *Salvia hispanica* Extract in Diabetic Rat. Cardiovasc. Hematol. Agents Med. Chem..

[9] Song J., Kim Y.-S., Kim L., Park H.J., Lee D., Kim H. (2019). Anti-Obesity Effects of the Flower of Prunus persica in High-Fat Diet-Induced Obese Mice. Nutrients.

[10] Bravo L. (1998). Polyphenols: Chemistry, Dietary Sources, Metabolism, and Nutritional Significance. Nutr. Rev..

[11] Hagerman A.E., Riedl K.M., Jones G.A., Sovik K.N., Ritchard N.T., Hartzfeld P.W., Riechel T.L. (1998). High Molecular Weight Plant Polyphenolics (Tannins) as Biological Antioxidants. J. Agric. Food Chem..

[12] Williams R.J., Spencer J.P., Rice-Evans C. (2004). Flavonoids: Antioxidants or signalling molecules?. Free Radic. Biol. Med..

[13] Pastor-Villaescusa B., Sanchez Rodriguez E., Rangel-Huerta O.D., del Moral A.M., Aguilera García C.M. (2018). Chapter 11—Polyphenols in Obesity and Metabolic Syndrome. Obesity: Oxidative Stress and Dietary Antioxidants.

[14] Cho A.-S., Jeon S.-M., Kim M.-J., Yeo J., Seo K.-I., Choi M.-S., Lee M.-K. (2010). Chlorogenic acid exhibits anti-obesity property and improves lipid metabolism in high-fat diet-induced-obese mice. Food Chem. Toxicol..

[15] Vasileva L.V., Savova M.S., Tews D., Wabitsch M., Georgiev M.I. (2021). Rosmarinic acid attenuates obesity and obesity-related inflammation in human adipocytes. Food Chem. Toxicol..

[16] Zúñiga-López M.C., Maturana G., Campmajó G., Saurina J., Núñez O. (2021). Determination of Bioactive Compounds in Sequential Extracts of Chia Leaf (*Salvia hispanica* L.) Using UHPLC-HRMS (Q-Orbitrap) and a Global Evaluation of Antioxidant In Vitro Capacity. Antioxidants.

[17] Cahill J.P. (2004). Genetic diversity among varieties of Chia (*Salvia hispanica* L.). Genet. Resour. Crop Evolut..

[18] Jamboonsri W., Phillips T.D., Geneve R.L., Cahill J.P., Hildebrand D.F. (2012). Extending the range of an ancient crop, *Salvia hispanica* L.—A new ω3 source. Genet. Resour. Crop. Evol..

[19] Reyes-Caudillo E., Tecante A., Valdivia-López M. (2008). Dietary fibre content and antioxidant activity of phenolic compounds present in Mexican chia (*Salvia hispanica* L.) seeds. Food Chem..

[20] Peiretti P., Gai F. (2009). Fatty acid and nutritive quality of chia (*Salvia hispanica* L.) seeds and plant during growth. Anim. Feed. Sci. Technol..

[21] Martínez-Cruz O., Paredes-López O. (2014). Phytochemical profile and nutraceutical potential of chia seeds (*Salvia hispanica* L.) by ultra high performance liquid chromatography. J. Chromatogr. A.

[22] Kulczyński B., Kobus-Cisowska J., Taczanowski M., Kmiecik D., Gramza-Michałowska A. (2019). The Chemical Composition and Nutritional Value of Chia Seeds—Current State of Knowledge. Nutrients.

[23] Kiani M., Rabiee N., Bagherzadeh M., Ghadiri A.M., Fatahi Y., Dinarvand R., Webster T.J. (2020). High-gravity-assisted green synthesis of palladium nanoparticles: The flowering of nanomedicine. Nanomed. Nanotechnol. Biol. Med..

[24] Elshafie H.S., Aliberti L., Amato M., De Feo V., Camele I. (2018). Chemical composition and antimicrobial activity of chia (*Salvia hispanica* L.) essential oil. Eur. Food Res. Technol..

[25] Amato M., Caruso M.C., Guzzo F., Galgano F., Commisso M., Bochicchio R., Labella R., Favati F. (2015). Nutritional quality of seeds and leaf metabolites of Chia (*Salvia hispanica* L.) from Southern Italy. Eur. Food Res. Technol..

[26] Liang W., Menke A.L., Driessen A., Koek G.H., Lindeman J.H., Stoop R., Havekes L.M., Kleemann R., van den Hoek A.M. (2014). Establishment of a General NAFLD Scoring System for Rodent Models and Comparison to Human Liver Pathology. PLoS ONE.

[27] Jervis G., Ladue J.S., Wroblewski F. (1956). The diagnostic, prognostic and epidemiologic significance of serum glutamic oxaloacetic transaminase (SGO-T) alterations in acute hepatitis. Ann. Intern. Med..

[28] Simoes I.C., Janikiewicz J., Bauer J., Karkucinska-Wieckowska A., Kalinowski P., Dobrzyń A., Wolski A., Pronicki M., Zieniewicz K., Dobrzyń P. (2019). Fat and Sugar—A Dangerous Duet. A Comparative Review on Metabolic Remodeling in Rodent Models of Nonalcoholic Fatty Liver Disease. Nutrients.

[29] Ngo Y.L., Lau C.H., Chua L.S. (2018). Review on rosmarinic acid extraction, fractionation and its anti-diabetic potential. Food Chem. Toxicol..

[30] Russo B., Picconi F., Malandrucco I., Frontoni S. (2019). Flavonoids and Insulin-Resistance: From Molecular Evidences to Clinical Trials. Int. J. Mol. Sci..

[31] La Fuente F.P.-D., Nocetti D., Sacristán C., Ruiz P., Guerrero J., Jorquera G., Uribe E., Bucarey J.L., Espinosa A., Puente L. (2020). *Physalis peruviana* L. Pulp Prevents Liver Inflammation and Insulin Resistance in Skeletal Muscles of Diet-Induced Obese Mice. Nutrients.

[32] Feng G., Byrne C.D., Targher G., Wang F., Zheng M. (2022). Ferroptosis and metabolic dysfunction-associated fatty liver disease: Is there a link?. Liver Int..

[33] Van De Wier B., Koek G.H., Bast A., Haenen G. (2017). The potential of flavonoids in the treatment of non-alcoholic fatty liver disease. Crit. Rev. Food Sci. Nutr..

[34] Mu H.-N., Zhou Q., Yang R.-Y., Tang W.-Q., Li H.-X., Wang S.-M., Li J., Chen W.-X., Dong J. (2021). Caffeic acid prevents non-alcoholic fatty liver disease induced by a high-fat diet through gut microbiota modulation in mice. Food Res. Int..

[35] Kühn H., Borchert A. (2002). Regulation of enzymatic lipid peroxidation: The interplay of peroxidizing and peroxide reducing enzymes. Free Radic. Biol. Med..

[36] Vidyashankar S., Varma R.S., Patki P.S. (2013). Quercetin ameliorate insulin resistance and up-regulates cellular antioxidants during oleic acid induced hepatic steatosis in HepG2 cells. Toxicol. Vitr..

[37] Liu Y., Wang D., Zhang D., Lv Y., Wei Y., Wu W., Zhou F., Tang M., Mao T., Li M. (2011). Inhibitory Effect of Blueberry Polyphenolic Compounds on Oleic Acid-Induced Hepatic Steatosis in Vitro. J. Agric. Food Chem..

