# Supplementary material for: Evaluation of the Effects of Chia (Salvia hispanica L.) Leaves Ethanolic Extracts Supplementation on Biochemical and Hepatic Markers on Diet-Induced Obese Mice
Source: Antioxidants (Basel). 2023 May 17;12(5):1108. doi: 10.3390/antiox12051108 (PMC10215631; doi:10.3390/antiox12051108)
Supplement: Supplementary file 1 [file antioxidants-12-01108-s001.zip › antioxidants-2378559-supplementary.pdf]

Supplementary material.

A

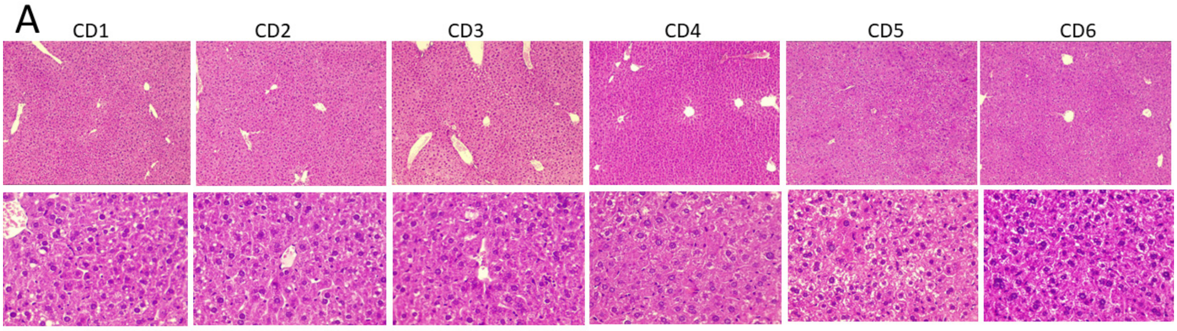

B

	CD1	CD2	CD3	CD4	CD5	CD6
Macroesteatosis (% / score)	4/0	3/0	4/0	2/0	4/0	3/0
Microesteatosis (% /score)	6/1	5/0	3/0	4/0	8/1	6/1
Hipertrophy	0	0	0	0	0	0
Immflamatory focus	0	0	0	0	0	0
Score (X/12)	1	0	0	0	1	1

Figure S1. Representative bright field image of an H&E stained liver section. Images are representatives of each animal from control group (n=6). A. Upper panel shows 10X and bottom panel shows 40X magnification, respectively. B. Calculated score for steatosis expressed of a 12 total score.

A

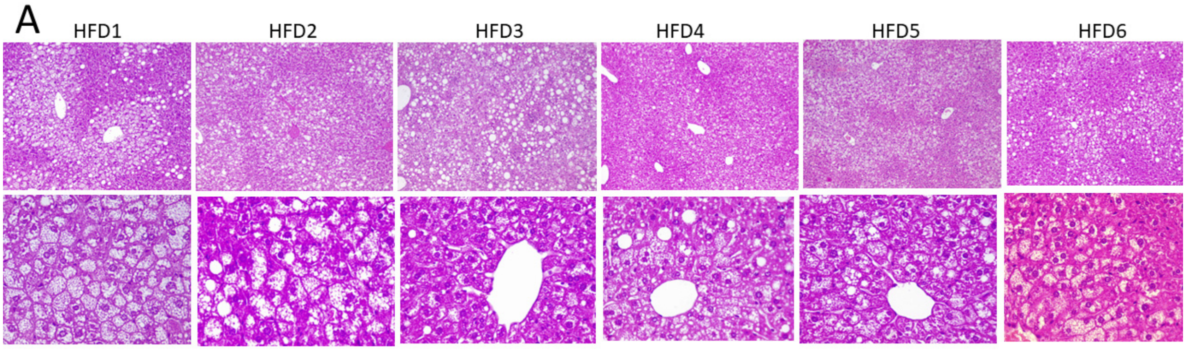

B

	HFD1	HFD2	HFD3	HFD4	HFD5	HFD6
Macroesteatosis (% / score)	8/1	67/1	22/1	10/1	21/1	16/1
Microesteatosis (% /score)	38/2	45/2	43/2	4/2	33/2	28/1
Hipertrophy	2	2	2	2	2	2
Immflamatory focus	0	1	1	0	0	0
Score (X/12)	5	6	6	5	5	4

Figure S2. Representative bright field image of an H&E stained liver section. Images are representatives of each animal from HFD group (n=6). A. Upper panel shows 10X and bottom panel shows 40X magnification, respectively. B. Calculated score for steatosis expressed of a 12 total score.

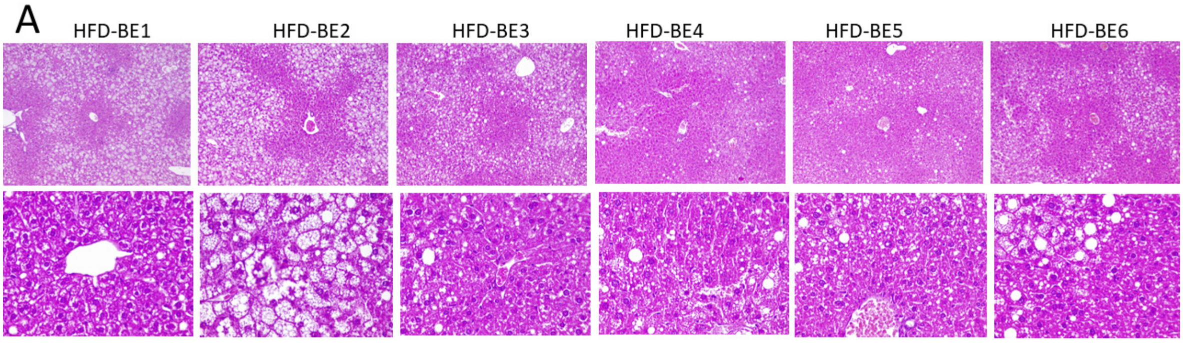

B

	HFD1	HFD2	HFD3	HFD4	HFD5	HFD6
Macroesteatosis (% / score)	4/0	5/1	8/1	4/0	16/1	10/1
Microesteatosis (% /score)	25/1	37/2	40/2	15/1	20/1	38/2
Hipertrophy	1	0	0	0	0	0
Immflamatory focus	1	0	0	0	0	0
Score (X/12)	3	3	3	1	2	3

Figure S3. Representative bright field image of an H&E stained liver section. Images are representatives of each animal from HFD-BE group (n=6). A. Upper panel shows 10X and bottom panel shows 40X magnification, respectively. B. Calculated score for steatosis expressed of a 12 total score.

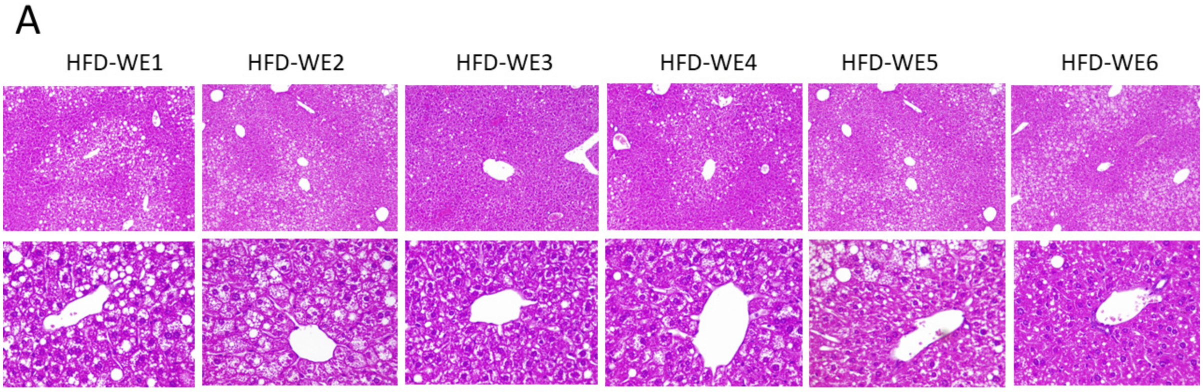

B

	HFD1	HFD2	HFD3	HFD4	HFD5	HFD6
Macroesteatosis (% / score)	18/1	10/1	10/1	15/1	7/1	20/1
Microesteatosis (% /score)	21/1	33/2	25/1	17/1	33/2	23/1
Hipertrophy	0	1	0	0	1	0
Immflamatory focus	0	0	0	0	0	0
Score (X/12)	2	4	2	2	4	2

Figure S4. Representative bright field image of an H&E stained liver section. Images are representatives of each animal from HFD-WE group (n=6). A. Upper panel shows 10X and bottom panel shows 40X magnification, respectively. B. Calculated score for steatosis expressed of a 12 total score.

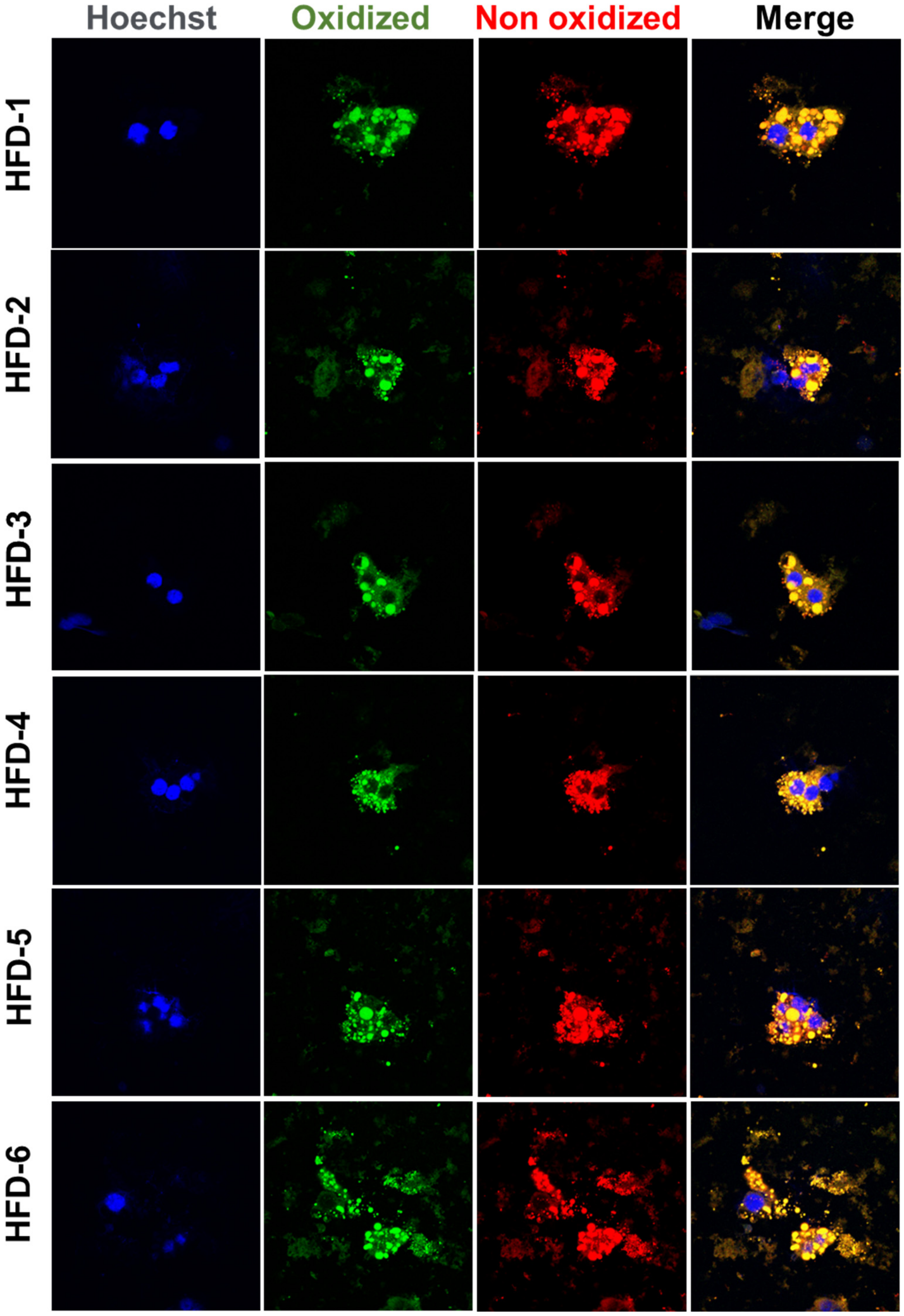

Figure S5. Representative confocal images of an BODIPY-c11 stained isolated hepatocytes. Images are representatives of each animal from HFD group (n=6). 40X magnification.

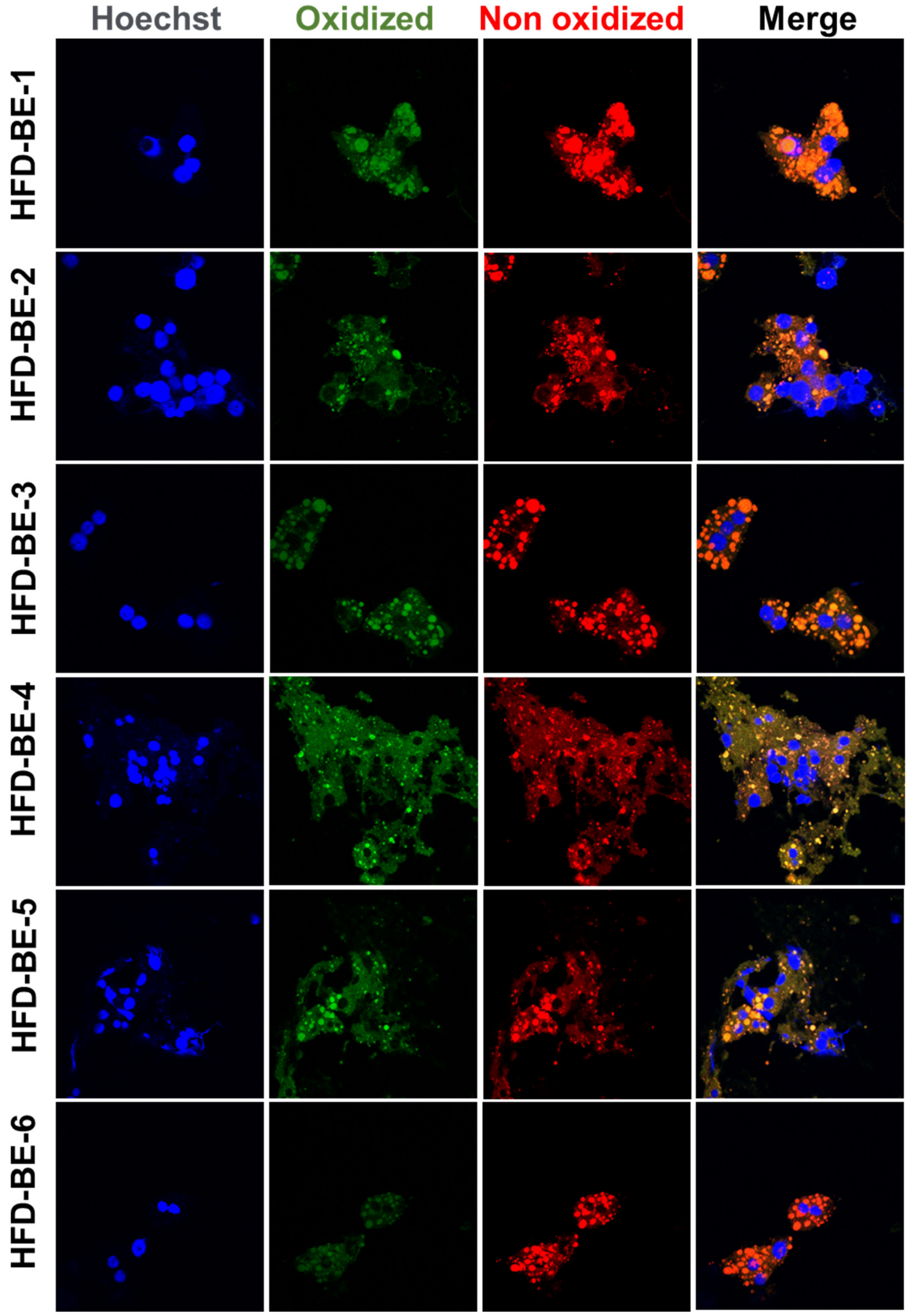

Figure S6. Representative confocal images of an BODIPY-c11 stained isolated hepatocytes. Images are representatives of each animal from HFD-BE group (n=6). 40X magnification.

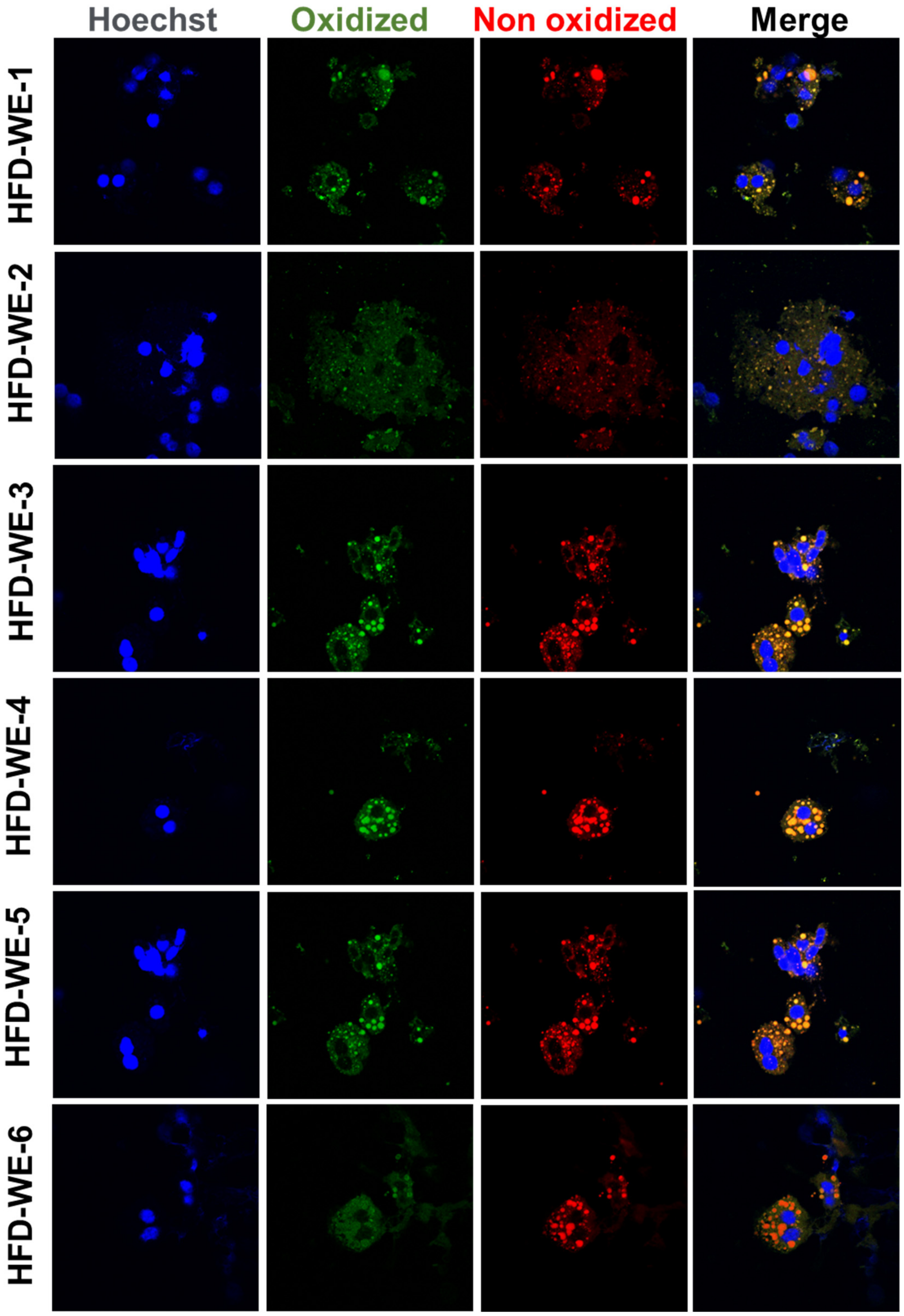

Figure S7. Representative confocal images of an BODIPY-c11 stained isolated hepatocytes. Images are representatives of each animal from HFD-WE group (n=6). 40X magnification
